# Generalization of three-dimensional golden-angle radial acquisition to reduce eddy current artifacts in bSSFP CMR imaging

**DOI:** 10.1007/s10334-020-00859-z

**Published:** 2020-06-26

**Authors:** Alexander Fyrdahl, Karen Holst, Kenneth Caidahl, Martin Ugander, Andreas Sigfridsson

**Affiliations:** 1 Department of Clinical Physiology, Karolinska University Hospital, and Karolinska Institutet, Stockholm, Sweden; 2 Sahlgrenska Academy, Gothenburg University, and Västra Götaland Region, Sahlgrenska University Hospital, Gothenburg, Sweden; 3 The Kolling Institute, Royal North Shore Hospital, and Northern Clinical School, Sydney Medical School, University of Sydney, Sydney, Australia

**Keywords:** Eddy currents, Golden-angle, CMR

## Abstract

**Purpose:**

We propose a novel generalization of the three-dimensional double-golden-angle profile ordering, which allows for whole-heart volumetric imaging with retrospective binning and reduced eddy current artifacts.

**Methods:**

A novel theory bridging the gap between the three-dimensional double golden-angle trajectory, and the two-dimensional tiny-golden-angle trajectory is presented. This enables a class of double golden-angle profile orderings with a smaller angular distance between successive *k*-space readouts. The novel profile orderings were evaluated through simulations, phantom experiments, and in vivo imaging. Comparisons were made to the original double-golden-angle trajectory. Image uniformity and off-resonance sensitivity were evaluated using phantom measurements, and qualitative image quality was assessed using in vivo images acquired in a healthy volunteer.

**Results:**

The proposed theory successfully reduced the angular step while maintaining image uniformity after binning. Simulations revealed a slow degradation with decreasing angular steps and an increasing number of physiological bins. The phantom images showed a definite improvement in image uniformity and increased robustness to off-resonance, and in vivo imaging corroborated those findings.

**Conclusion:**

Reducing the angular step in cardio-respiratory-binned golden-angle imaging shows potential for overcoming eddy current-induced image artifacts associated with 3D golden-angle radial imaging.

## Introduction

Cardiovascular magnetic resonance imaging (CMR) is seeing increased use worldwide and has become the reference standard in diagnosing left ventricular systolic function. However, due to technical limitations, imaging has typically been performed in multiple two-dimensional (2D) slices, where each slice is acquired during breath-holding. Despite using end-expiratory breath-holding for improved repeatability, residual misalignment between adjacent slices may affect the accuracy of the measurement [1]. Furthermore, breath-holding puts unnecessary stress on the patient and discards the additional information to be derived from the interaction between preload and ventricular filling [2]. Previous studies have shown that we can not only do without breath-holding but also acquire the entire heart in a single three-dimensional (3D) image volume, while the patient breathes freely [3]. Many proposed strategies for free-breathing whole-heart imaging are based on radial trajectories that provide higher SNR per unit time compared to Cartesian strategies, as well as the flexibility of post hoc decisions regarding spatial and temporal resolution, integrated motion detection [4], and the ability to perform data sorting. Particularly attractive in this regard is the golden-angle radial trajectory [5], which was originally proposed for 2D, but can be expanded to 3D either by stacking multiple 2D golden-angle trajectories on top of each other to form a *stack of stars* [6] or by constructing a trajectory with readouts originating at the surface of a sphere [7], sometimes called Koosh ball sampling. The latter has shown great potential for dynamic breast [8] and free-breathing whole-heart cardiac MRI [9]. It provides inherent isotropic resolution, which facilitates multiplanar reformatting and simplifies planning. The *golden property,* which is characteristic of all golden-angle trajectories, can be expressed as simultaneously seeking to maximize the distance between successive readouts in time while minimizing the distance between adjacent readouts in *k*-space. This ensures even *k*-space coverage for an arbitrary number of readouts, but it comes at the cost of eddy current-induced image artifacts when used with a balanced steady-state free precession (bSSFP) readout [10]. Despite this disadvantage, bSSFP imaging is widely used in CMR due to enabling rapid imaging and excellent blood-myocardium contrast. The bSSFP method relies on the spin dephasing to be constant between successive repetition times (TR), so a transversal equilibrium can be established over the two-TR phase cycle [11]. However, the eddy current response cannot be assumed to be isotropic, so angular differences between successive readouts results in TR-to-TR differences in dephasing due to eddy currents. This violates the assumption of constant dephasing and perturbs the transversal spin equilibrium [10], resulting in image artifacts. Previous work has proposed the *spiral phyllotaxis* trajectory, which is robust against eddy current effects and exhibits excellent *k*-space uniformity for consecutive readouts [12]. However, functional cine CMR generally necessitates sorting of readouts into different cardiac phases, so-called physiological binning, to resolve the cardiac cycle temporally. Binning a continuous spiral will leave holes in the sampling pattern. Therefore, the trajectory must be interleaved to enable binning. The sampling pattern can be divided into shorter spiral arms, which self-arrange such that the distance between readouts is minimized. However, the distance between each readout is simultaneously determined by the number of readouts and the number of interleaves, which makes the trajectory characteristics critically dependent on the acquisition scheme. Another way to express this is that the spiral phyllotaxis pattern is generated by a closed set of points that must be selected a priori.

In the present work, a unifying theory of the golden-angle trajectory design based on generalized Fibonacci sequences is presented, and we propose a novel 3D golden-angle trajectory in analogy to previously proposed 2D tiny golden-angle trajectory [13]. We analyze the performance of the novel trajectory with respect to the image and *k*-space uniformity after physiological binning, both in vitro and in vivo.

## Theory

### Review of an existing generalization of the golden angle in 2D

For 2D golden-angles [5], there exists an infinite series of angles with approximately golden properties [13]. The *N*th generalized conjugate golden ratio can be derived through a simple geometric construction:

1 $$\phi_{N} : = \frac{1}{{N - 1 + \frac{1}{\phi }}} ,$$

which can be understood in terms of a continued fraction, where all partial quotients, except for the first, is unitary, i.e., $\phi_{N} = [1; \,N,\overline{1]}$, making it hard to approximate as a ratio of rational numbers. The best rational approximation of the golden ratio is the ratio of two consecutive Fibonacci numbers. This holds true for generalized Fibonacci series as well, meaning that the tiny golden ratios can be approximated at the limit of a generalized Fibonacci series defined as $G^{N} \left( n \right) = G^{N} \left( {n - 1} \right) + G^{N} \left( {n - 2} \right)$ for $n > 0$ with initial conditions $G^{N} \left( 1 \right) = 1, G^{N} \left( 2 \right) = N$, where

2 $$\mathop {\lim }\limits_{n \to \infty } \frac{{G^{1} \left( n \right)}}{{G^{N} \left( {n + 1} \right)}} = \phi_{N} .$$

Because the generalized golden-angles can be seen as a continuation of the original and the small golden-angle, which in this formalism is given by $N \ge 2$, they were named *tiny* golden-angles [13].

### A novel generalization of the golden angle in 3D

The concept of the golden ratio can be generalized to higher dimensions, where the high dimensional analogies to the conjugate golden ratio have been referred to as the golden means [14]. In 3D, the two golden means can be defined as $\phi_{1} = 0.4656$ and $\phi_{2} = 0.6823$. These can be mapped onto the surface of a sphere using the following area-preserving transform:

3 $$\alpha = \cos^{ - 1} \left\{ {m\phi_{1} } \right\} \,{\text{and}}\, \beta = 2\pi \left\{ {m\phi_{2} } \right\},$$

where $\alpha$ is the polar angle, $\beta$ is the azimuthal angle, { } denotes the modulo of 1 and $m$ counts the acquisition ordering [8]. This three-dimensional scheme will be referred to as the double golden-angle ordering. It is noted that $\left\{ {m\phi_{1} } \right\}$ and $\left\{ {m\phi_{2} } \right\}$ from Eq. (1) can be considered Kronecker-type sequences [15]. As such, any *irrational* values of $\phi_{1}$ and $\phi_{2}$ ($\phi_{1} \ne \phi_{2} )$ would generate a low-discrepancy Kronecker sequence. To select suitable values, a geometrical construction similar to Eq. 2 can be used, which also holds for 3D golden means

4 $$\phi_{N,2} : = \frac{1}{{N - 1 + \frac{1}{{\phi_{2} }}}}\, {\text{and}} \,\phi_{N,1} : = \frac{{\phi_{N,2} }}{{1 + \phi_{1} }},$$

where $\phi_{1} = 0.4656$ and $\phi_{2} = 0.6823$ are the original golden means. Likewise, the golden means can be generated from the generalized Fibonacci series $H^{N} \left( n \right) = H^{N} \left( {n - 1} \right) + H^{N} \left( {n - 3} \right)$ for $n \ge 0$ and with initial conditions $H^{N} \left( 0 \right) = 0, H^{N} \left( 1 \right) = 1, H^{N} \left( 2 \right) = N$, where

5 $$\mathop {\lim }\limits_{n \to \infty } \frac{{H^{1} \left( n \right)}}{{H^{N} \left( {n + 1} \right)}} = \phi_{N,2} \,{\text{and }}\, \mathop {\lim }\limits_{n \to \infty } \frac{{H^{1} \left( n \right)}}{{H^{N} \left( {n + 2} \right)}} = \phi_{N,1} .$$

**Fig. 1:**
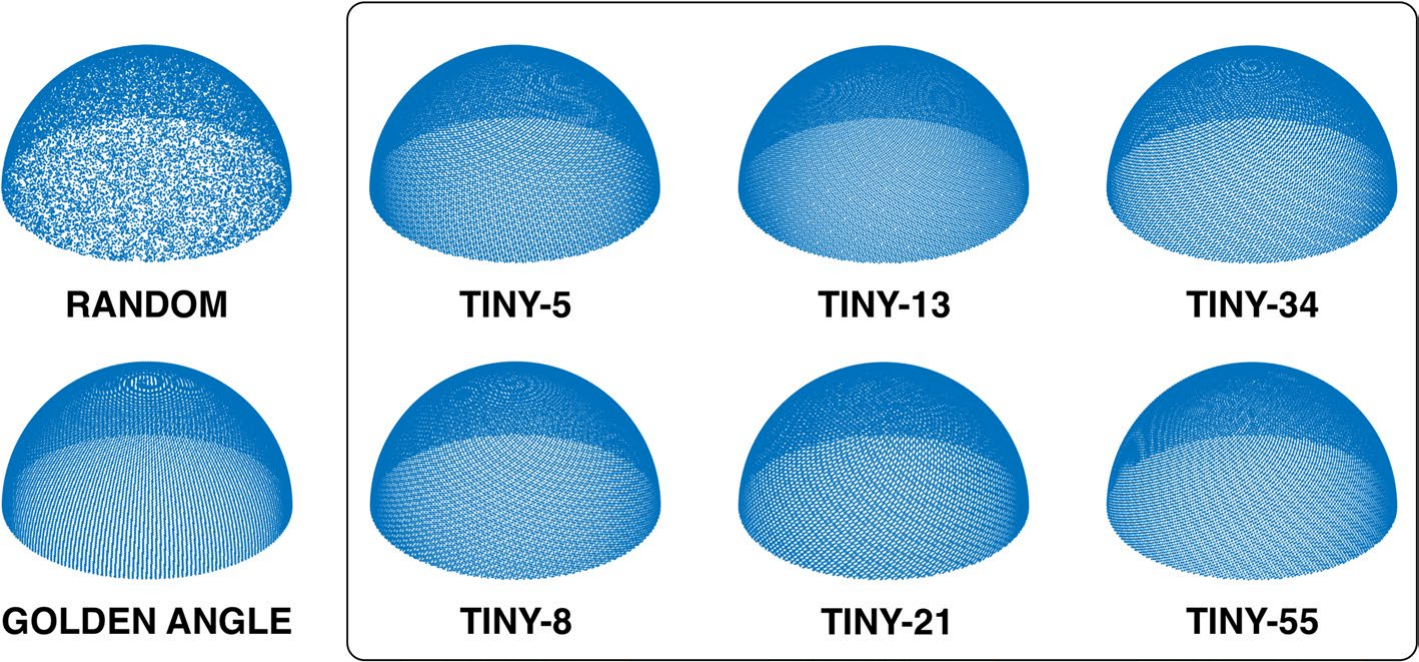
Graphical representation of the different trajectories. The golden and tiny trajectories all are perceptually uniform compared to the random trajectory. All trajectories were calculated with the same number of sample points, *N* = 28,657

Due to the similarity between the 2D generalized golden ratios and the 3D generalized golden means, we will refer to the generalized golden means with $N \ge 2$ as tiny golden means. Using this generalization, *N* = 1 represents the original golden means. Figure 1 shows a selection of profile orderings generated from the tiny golden means.

### Clustered, random, quasi-random or regular

A statistical approach to quantifying the discrepancy of a distribution is the Clustered-Random-Regular (CRR) continuum [16]. If the points of the distribution are completely randomly distributed, the CRR is 1. If the points are clustered together, this will result in a CRR < 1. A regular distribution, on the other hand, will have a CRR between 1 and 2, and if the points fall on the intersections of a rectilinear grid, the CRR is 2. For a set of *P* points, the mean nearest neighbor distance can be calculated as

6 $$r_{0} = \frac{1}{P}\mathop \sum \limits_{i \ne j}^{P} \hbox{min} \left( {u_{i,j} } \right),$$

where $u_{i,j}$ is the distance between points $i$ and $j$. For a random distribution, the expectation value of the mean nearest neighbor distance can be assumed to be

7 $$r_{e} = \frac{1}{2}\sqrt {\frac{1}{P}} .$$

The CRR is then given by the ratio of Eqs. 6 and 7:

8 $${\text{CRR}} = \frac{{r_{0} }}{{r_{e} }} = 2\frac{{\mathop \sum \nolimits_{i \ne j}^{P} \hbox{min} \left( {u_{i,j} } \right)}}{\sqrt P }.$$

## Methods

### Nomenclature

When referring to a radial profile order generated from a certain Fibonacci series, Tiny-$N$ was used as the name for the radial profile order defined by the tiny golden means $\phi_{N,1}$ and $\phi_{N,2}$, generated from the generalized Fibonacci series $H^{N}$.

### Step size

The magnitude of the eddy current-induced effects on the images is expected to depend on the step size between successive readout profiles. Due to the non-linearity of the mapping operation onto the sphere, the distance between successive readouts will vary. Furthermore, the distance in *k*-space is dependent on voxel size. Thus, the angles between successive readout profiles were used as a metric of step size and calculated as

9 $$\angle = \cos^{ - 1} \left( {\frac{{v_{i} \cdot v_{i + 1} }}{{\left| {v_{i} } \right|\left| {v_{i + 1} } \right|}}} \right),$$

where ${\text{v}}_{i} = \left( {x_{i} ,y_{i} ,z_{i} } \right)$ was the vector between the center of the trajectory sphere and the origin of the readout at the surface of the sphere. The mean angle between successive readouts was reported for the conventional double golden-angle profile ordering, Tiny-5, Tiny-8, Tiny-13, Tiny-21, Tiny-34, and Tiny-55. N was chosen as Fibonacci numbers to achieve approximately equidistant angular steps.

### Clustered, random, quasi-random or regular

The CRR of the spoke distribution was calculated for the conventional double golden-angle profile ordering, Tiny-5, Tiny-8, Tiny-13, Tiny-21, Tiny-34, and Tiny-55 according to Eq. (8). The CRR was calculated for a number of spokes representing every Fibonacci number between 233 and 121,393. The average CRR value for each profile ordering was reported.

### Binning simulations

To investigate the robustness of the proposed tiny golden-angle profile orderings to physiological binning, numerical binning simulations were performed from recorded physiological data. ECG and respiratory motion signals were recorded in 8 healthy volunteers and used to simulate cardiac and respiratory binning of 126,478 spokes using the original double golden-angle profile ordering and the proposed Tiny-13 profile ordering. Physiological binning was performed for all possible combinations of 20 and 25 cardiac phases and 2, 4, and 6 respiratory phases. For each of the bins, spherical Voronoi diagrams [17] were calculated on the surface of the trajectory sphere and the standard deviation of the cell areas was used as a measure of *k*-space distribution. All simulations incorporated a self-navigation spoke that was discarded prior to the calculation of the Voronoi diagrams. The number of spokes and number of bins were chosen to match previous work using 3D radial acquisitions of the whole heart [4, 9, 18].

### Pulse sequence

A prototype radial bSSFP pulse sequence was implemented with a double golden-angle profile ordering, with a user-selectable profile ordering based on an integer *N* = 1 to 100 corresponding to the desired profile ordering. The implementation allowed for imaging in a free-running mode, where the entire readout was done sequentially, without pause following a standard α/2 − TR/2 preparation to establish a steady-state.

### Phantom image acquisition

Phantom image acquisitions were performed at 1.5 T (MAGNETOM Aera, Siemens Healthcare, Erlangen, Germany). A spherical water phantom filled with a nickel sulfate solution (NiSO_4_) was placed in the isocenter of the scanner. To mitigate transient temperature effects, the gradients were continuously cycled for 15 min prior to image acquisition. Gradient coil temperatures were continuously monitored. Relevant imaging parameters were as follows: voxel size 1.25 mm isotropic, TE/TR 1.6/3.2 ms, flip angle 70°, receiver bandwidth 1015 Hz/px, 92,314 radial spokes, corresponding to approximately two times oversampling with respect to the radial Nyquist limit. To investigate the off-resonance performance, a linear phase increment $\Delta \omega$, in addition to the $180^\circ$ bSSFP phase cycle, was added to each RF-pulse to generate a frequency offset corresponding to

10 $$\Delta f = \frac{\Delta \omega }{{2\pi \cdot {\text{TR}}}}$$

The off-resonance was adjusted from − 180 to + 180 degrees (− ½ TR to ½ TR). The order of the phantom experiments was randomized to avoid systematic errors. For visualization purposes, a bottle containing the same NiSO_4_ solution was imaged with the same imaging parameters, except that only 48,657 radial spokes were acquired, which corresponded to the radial Nyquist limit for the given image resolution.

### In vivo image acquisition

In vivo images were acquired at 1.5 T (MAGNETOM Aera, Siemens Healthcare, Erlangen, Germany). Relevant imaging parameters were as follows: voxel size 1.2 mm isotropic, TE/TR 1.6/3.2 ms, flip angle 50°, receiver bandwidth 1015 Hz/px, 131,072 radial spokes, corresponding to an acquisition time of approximately 7 min. A superior-inferior (SI) spoke was interleaved every 25th TR. Two acquisitions were performed, one with the originally proposed double golden-angle profile ordering and one with the Tiny-13 profile ordering, which was chosen based on the previous experiments. Both in vivo acquisitions were performed under equal conditions and in direct succession. No frequency adjustments were made between the two acquisitions.

### Image reconstruction

Phantom images were reconstructed by direct gridding using the gpuNUFFT toolbox [19]. In vivo images were reconstructed using the BART framework [20]. The SI spoke was extracted, and a Fourier transform was applied along the readout direction, resulting in a projection image in the SI direction of the body. Principal component analysis was performed along all coils to detect the dominating motion components. The detected principal components were then transformed into the frequency domain, and the component with the strongest frequency peak in the range of 0.1–0.7 Hz was selected to represent the respiratory motion. The cardiac cycles were extracted from the recorded patient ECG and the respiratory and cardiac cycles were normalized. A cardio-respiratory motion map was calculated as previously described [21] with 20 cardiac phases and 2 respiratory phases. Coil sensitivity maps were calculated using an adaptive filter method [22]. All image reconstructions were performed in MATLAB 2019b (MathWorks, Natick, MA, USA) running on a Linux workstation equipped with two 14-core Xeon CPUs (Intel, Santa Clara, CA. USA), 128 GB RAM and a QUADRO M6000 GPU with 24 GB VRAM (Nvidia, Santa Clara, CA, USA). Multiplanar reformatting was performed using an in-house-developed software.

### Assessment of image uniformity

The image uniformity in the spherical phantom was assessed by calculating the normalized average absolute deviation (NAAD) of the signal intensity. To reduce confounding noise effects, the images were convolved with a 3 × 3 × 3 cosine filter kernel. The NAAD was measured in a spherical volume of interest (VOI) that encompassed 75% of the phantom volume and was calculated as

11 $${\text{NAAD}} = 1 - \frac{{\mathop \sum \nolimits_{i = 1}^{N} \left| {s_{i} - \mu} \right|}}{\mu M}$$

where $s$ is the signal intensity in the volume, $\mu$ is the mean within the volume and M is the number of voxels within the VOI. A perfectly uniform image had a NAAD of 1 and a lower value indicated a lower degree of uniformity.

### Statistics

Continuous variables were reported as mean and standard deviation. Ordinal variables were reported as percentages. Normally distributed values, as determined by the Kolmogorov–Smirnov test, were compared using the paired or non-paired version of Student’s *t* test or suitable non-parametric counterparts such as the Wilcoxon signed-rank test as appropriate.

*P* < 0.05 was considered statistically significant. All statistical tests were performed in MATLAB 2019b (MathWorks, Natick, MA, USA).

## Results

### Step size

**Fig. 2:**
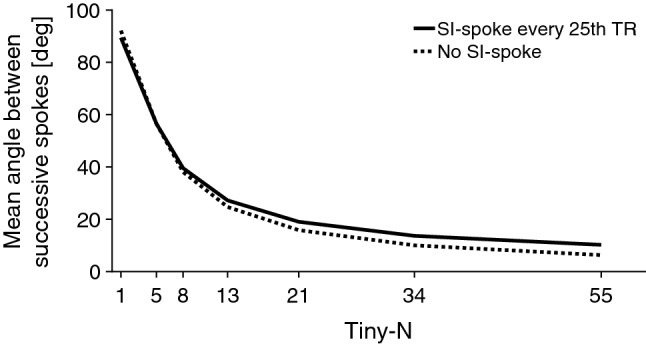
Mean angle between successive spokes (dashed line). The solid line represents the angular step with a superior-inferior spoke interspersed every 25th TR

The step size for each profile ordering is indicated in Fig. 2. The mean angle between successive spokes was 92 degrees for the conventional golden-angle profile ordering and decreased approximately exponentially. The Tiny-13 ordering chosen for analysis in this work had a mean angle of 25 degrees between successive spokes. When incorporating a navigator spoke with a fixed frequency, the mean angle decreased slightly for the original golden-angle ordering from 94 to 89 degrees, as the angle between any spoke on the hemisphere and the pole was never more than 90 degrees. For Tiny-13 the mean angle increased slightly from 25 to 27 degrees.

### Clustered, random, quasi-random or regular

**Fig. 3:**
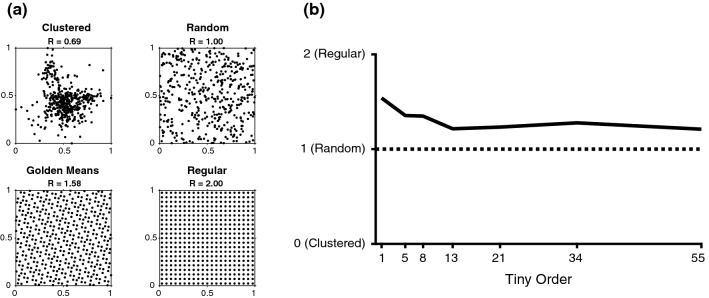
**a** Schematic illustration of the Clustered-Random-Regular (CRR) metric, together with the conventional golden-means distribution. The top left panel describes a clustered appearance, with CRR < 1. The top right appearance describes a random distribution with CRR = 1. The bottom left panel describes the golden-means distribution with CRR = 1.58 The bottom right panel describes a perfectly regular pattern, with CRR = 2. A quasi-random distribution is expected to fall in the range between 1 and 2. **b** CRR-values for a selection of tiny orders up to 55, the presented value is the average CRR for the range from 233 to 121,393 spokes

The schematic difference between a clustered, random, quasi-random and ordered set is displayed in Fig. 3a. The results from the CRR uniformity evaluation is shown in Fig. 3b. The highest CRR was found for the original golden-angle profile ordering with CRR = 1.5, and the lowest CRR was found for Tiny-13, where CRR = 1.2. All of the other tested profile orderings fell between 1.2 and 1.5. Tiny-13 was chosen for further testing, as it represented a worst-case scenario in terms of CRR.

### Binning simulations

**Fig. 4:**
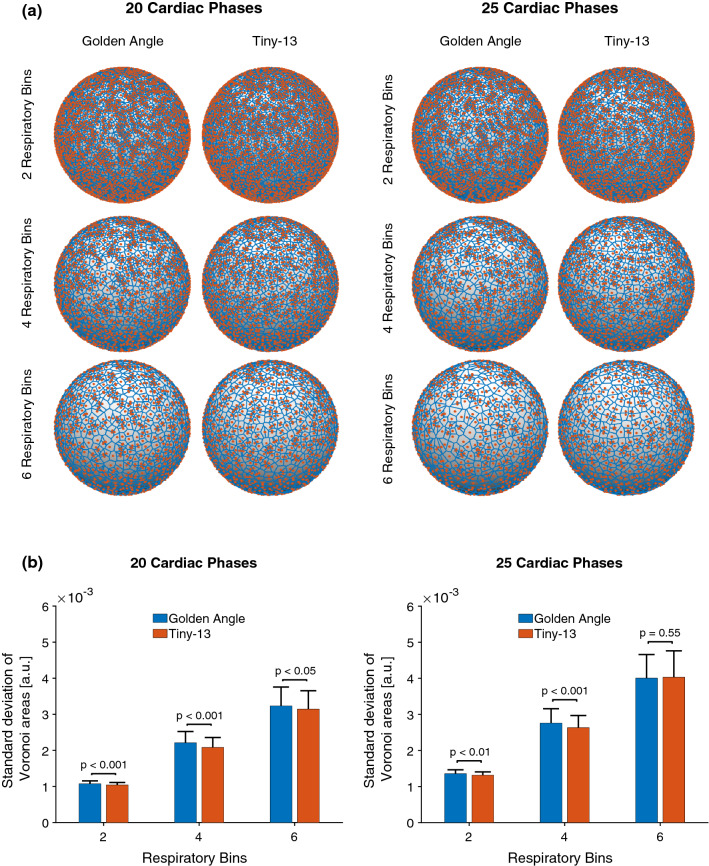
**a** Representative Voronoi diagrams used to assess the *k*-space uniformity after binning for the conventional double golden-angle and the proposed Tiny-13 profile ordering. Red points represent the origin or end of a readout spoke. Blue lines represent the boundaries of the Voronoi cells. **b** Bar plots describing the outcome of the binning simulations. The standard deviation of Voronoi cell areas was calculated for all combinations of respiratory and cardiac phases. Numbers are represented as mean ± SD. The blue bars represent the conventional double golden-angle profile ordering, whereas the red bars represent Tiny-13

The results of the binning simulations are shown in Fig. 4. Tiny-13 had a lower standard deviation of Voronoi cell areas for all tests except for 25 cardiac phases and 6 respiratory phases, where the difference did not reach statistical significance.

### Assessment of image uniformity

**Fig. 5:**
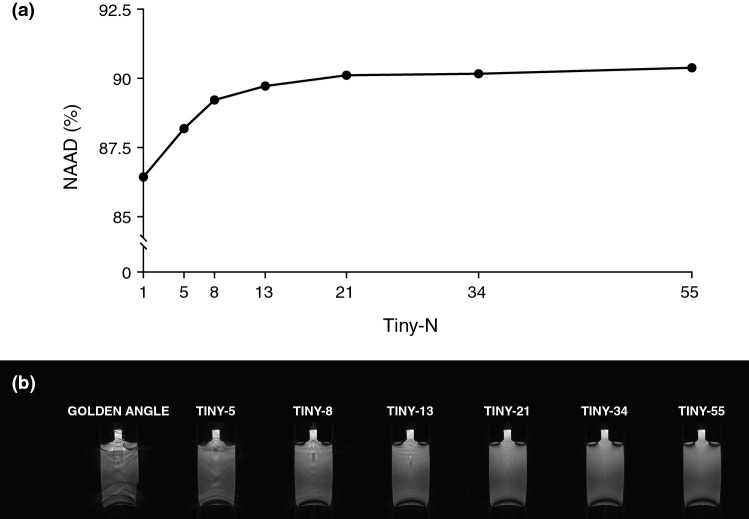
Phantom experiments demonstrate the improved image quality from the tiny golden profile orderings. **a** Normalized average absolute deviation (NAAD) was used to measure the image uniformity in a spherical phantom, which showed increasing uniformity with increasing tiny order. **b** Corresponding images of a bottle phantom

**Fig. 6:**
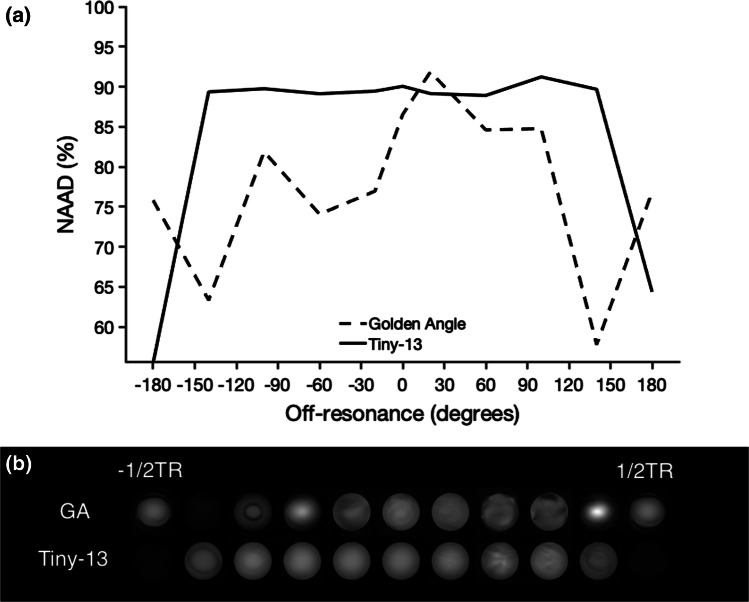
Phantom experiments indicating reduced sensitivity to off-resonance effects. **a** Normalized average absolute deviation (NAAD) is measured in a spherical volume of interest in a spherical phantom scanned with both the conventional three-dimensional golden-angle profile ordering, as well as the novel tiny golden-angle profile ordering Tiny-13. **b** Corresponding images are displayed, corroborating the numerical findings

Image uniformity results are presented in Fig. 5. The numerical results for the NAAD calculations are based on image acquisition using the spherical phantom. The uniformity increases with decreasing angular step. For conventional double golden-angle profile ordering, the NAAD was 86.4%. The NAAD increased up to Tiny-13, where NAAD was 89.7%. For higher orders, the difference diminished, and for Tiny-55 the NAAD was 90.4%. Panel B shows the qualitative comparisons for the same experiment but in a bottle phantom. Figure 6 shows the sensitivity to the off-resonance effect for the conventional double golden-angle profile ordering and Tiny-13. Here, it is evident that the signal varied considerably for the conventional profile ordering depending on the center frequency, whereas Tiny-13 showed the expected behavior with a uniform signal in the passband and signal cancellation in the stop band that occur at ± 180 degrees, as is to be expected for bSSFP imaging.

### Qualitative assessment of in vivo images

**Fig. 7:**
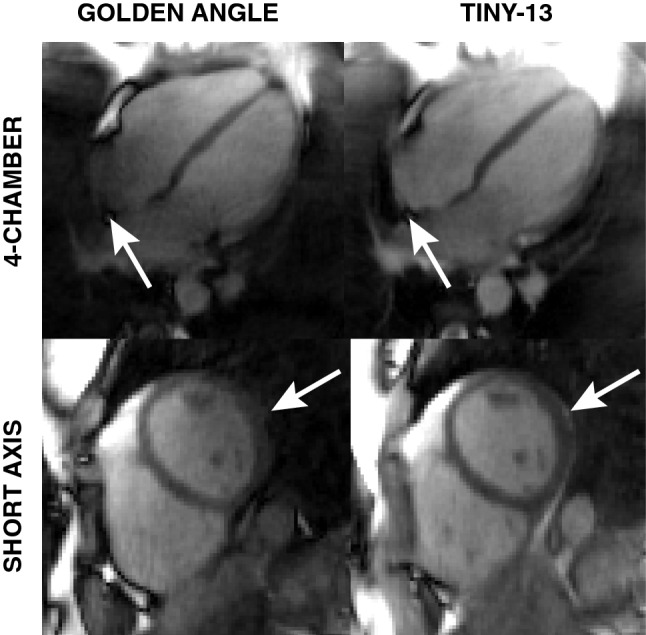
In vivo images show improved image quality with the reduced angular step, although the difference in eddy current-induced artifacts are not as pronounced as in the phantom experiments. With the conventional double golden-angle profile ordering there is an apparent signal loss in the right atrium and the epicardial fat (indicated by white arrows) which is not present with Tiny-13. Both images were acquired during free breathing and reconstructed from 126,478 spokes. The images represent the end-expiratory respiratory phase and the end-diastolic cardiac phase

Images from a human volunteer with the conventional double golden-angle and Tiny-13 profile orderings are shown in Fig. 7. Both the long- and short-axis images were extracted from the same image volume, for each profile ordering. There is an apparent signal loss in the right atrium as well as in the left lateral wall, and especially the epicardial fat signal is missing in the conventional double golden-angle acquisition. Both images were deemed to be of acceptable quality, with a slightly more homogenous signal in the Tiny-13 acquisition.

## Discussion

In this work, a novel, non-repeating, low-discrepancy *k*-space ordering that provides a 3D analogy to the 2D tiny golden-angles is presented.

By reducing the distance between successive readouts, the difference in the eddy current-dependent phase shift is expected to decrease. This, in turn, leads to a decrease in image artifacts, as evident by Fig. 5. For the conventional double golden-angle, considerable image artifacts were visible in the bottle, but the artifacts gradually disappeared as the angular step decreased. At Tiny-13, there were minimal residual artifacts, and for Tiny-21, Tiny-34, and Tiny-55 the difference was barely appreciable.

Figure 6 suggests improved stability and reduced sensitivity to off-resonance effects using the proposed profile ordering. The improvement in vivo was less dramatic than in the phantom measurements, but there is an appreciable improvement to the signal homogeneity in the right atrium and in the lateral wall, as indicated by Fig. 7.

A limitation of the current work is that it is not trivial to determine when the reduced angular methods will fail. In 2D golden-angle imaging, the angles add up to a full revolution after just 4 spokes. To successfully reconstruct an image volume, the spoke distribution of the sphere must be somewhat uniform. A loose constraint would be to assume that a similar number of spokes should originate in all octants of the three-dimensional *k*-space. However, this is a moving target, and it is not possible to define a limit when this becomes a real problem. The problem is somewhat mitigated by the fact that the Nyquist criterion is much higher for three-dimensional radial *k*-space compared to two-dimensional *k*-space. This may be part of the explanation as to why the difference between the Tiny-13 and conventional double golden-angle binning uniformity decreased as the number of bins increased. We expect there to be a minimum number of spokes needed for successful binning, and that minimum will increase with increasing tiny order. Considering these factors, we found Tiny-13 to be a reasonable candidate for the in vivo testing, as it balances the objectives of reduced angular step while still maintaining sufficient *k*-space coverage to allow for successful binning within a reasonable scan time. Using a higher tiny order will reduce the ability to perform the physiological binning necessary for cardiac imaging, resulting in worse *k*-space uniformity, unless the acquisition time is simultaneously increased.

The results in Figs. 1 and 3 suggest that the proposed Tiny profile ordering has a more uniform distribution than simply using random angles. Figure 7 shows that the tiny golden-angle approach does not negatively impact the ability to perform physiological binning, and can be used to enable cardiac- and respiratory resolved three-dimensional whole heart imaging. The efficacy of our novel generalization is supported by the idea that the conventional golden-angle profile orderings will provide an approximate uniform sampling for an arbitrary number of spokes. Whereas there are many strategies to three-dimensional radial imaging, some of which rely on a predefined lattice into which the sample points must fit, and hence, the number of sample points must be defined a priori. A more challenging task is to define a low-discrepancy set that maintains desired properties for effective and free-running *k*-space sampling while allowing for the number of sample points per lattice to be determined post hoc. We have proposed a natural method to derive such a set and tested it under a number of conditions. Whereas the results are promising, further testing in more subjects is warranted.

## Conclusion

The tiny golden-angle scheme was successful in reducing the angular step in cardio-respiratory-binned golden-angle imaging, and shows great potential for overcoming some of the limitations associated with 3D golden-angle radial imaging, in particular related to eddy current-induced image artifacts.
